# Predictive value of p53, Ki67 and TLR5 in neoplastic progression of Barrett’s esophagus: a matched case–control study

**DOI:** 10.1007/s00428-022-03340-5

**Published:** 2022-05-26

**Authors:** Olli Helminen, Jukka Melkko, Juha Saarnio, Eero Sihvo, Teijo Kuopio, Pasi Ohtonen, Joonas H. Kauppila, Tuomo J. Karttunen, Heikki Huhta

**Affiliations:** 1 Surgery Research Unit, Cancer and Translational Medicine Research Unit, Medical Research Center Oulu, University of Oulu and Oulu University Hospital, N90014 University of Oulu , PO-box 5000, Oulu, Finland; 2 Department of Surgery, Central Finland Central Hospital, Jyväskylä, Finland; 3 Department of Pathology, Central Finland Central Hospital, Jyväskylä, Finland; 4 Department of Biological and Environmental Science, University of Jyväskylä, Jyväskylä, Finland; 5 Division of Operative Care, Oulu University Hospital, Oulu, Finland; 6 Upper Gastrointestinal Surgery, Department of Molecular Medicine and Surgery, Karolinska Institutet, Stockholm, Sweden

**Keywords:** Barrett’s esophagus, Esophageal adenocarcinoma, Dysplasia marker, Immunohistochemistry, Surveillance, p53, Ki67, TLR5

## Abstract

Barrett’s esophagus progresses to high-grade dysplasia or cancer along the well-established metaplasia-dysplasia-adenocarcinoma sequence. The aim of this study was to evaluate the value of p53, Ki67, and toll-like receptor 5 (TLR5) in prediction of malignant progression of Barrett’s metaplasia and low-grade dysplasia. This was a retrospective matched case–control study based on Northern and Central Finland population. Patients diagnosed with esophageal high-grade dysplasia or adenocarcinoma were included. From these patients, all previous endoscopy samples were obtained along with original diagnostic HE-slides and clinical data. Age- and sex-matched patients with non-progressing Barrett’s metaplasia and low-grade dysplasia confirmed with follow-up endoscopies were used as controls. Two gastrointestinal pathologist re-reviewed all original HE-slides, and newly made sections to confirm representative tissue material blinded from clinical data. p53, Ki67, and TLR5 were immunohistochemically stained. Final cohort included 45 patients with progressive Barrett’s metaplasia (*n* = 21) or low-grade dysplasia (*n* = 24), and 92 patients with non-progressive Barrett’s metaplasia (*n* = 52) or low-grade dysplasia (*n* = 40). In Barrett’s metaplasia, aberrant p53 expression was observed in 6% of samples in progressors and 0% in non-progressors. In low-grade dysplasia, aberrant p53 was seen in 56% of samples in progressors and 17% in non-progressors (Odd’s ratio 6.7, 95% CI 1.8–24.6). Ki67 or TLR5 showed no association with disease progression. In this matched case–control study, p53 expression associated with a high risk of malignant progression in Barrett’s low-grade dysplasia. Routine staining of p53 is indicated in expert confirmed low-grade dysplasia.

## Introduction

Esophageal adenocarcinoma is characterized by increasing incidence and poor prognosis [1, 2], preceded by Barrett’s esophagus, where normal squamous mucosa is replaced with intestinal-type metaplastic columnar epithelium [3]. Excess exposure to acid and bile results in chronic inflammation and tissue damage, proposedly leading to columnar transformation [4]. Increasing incidence of Barrett’s esophagus might be linked to an increase in the prevalence of obesity-related reflux disease [5, 6].

Barrett’s esophagus progresses to dysplasia or cancer along the well-established metaplasia-dysplasia-adenocarcinoma sequence [7]. This progression is, however, rare and the majority of patients will never progress beyond the metaplastic state [8, 9]. Despite the low individual risk, patients with Barrett’s esophagus carry 150 times greater adenocarcinoma risk than those without [8]. Surveillance practices of Barrett’s esophagus vary widely [10], but no randomized controlled evidence supports routine follow-up [11]. For better risk stratification in non-dysplastic Barrett’s esophagus predictive biomarkers are needed to justify early treatment or follow-up [12]. Even for low-grade dysplasia (LGD), there are limited methods for identifying patients at highest risk for malignant transformation to facilitate treatment and surveillance [12]. If low-grade dysplasia is agreed upon by two expert pathologists, the risk of dysplasia progression increases significantly, but agreement is often not reached [13]. Previous biomarker studies have focused primarily on p53 with promising results, p53 being used in routine diagnostics in some centers [14–17]. Novel sampling methods, including Cytosponge, for better risk stratification have been developed, but these are not yet in routine clinical use [18]. Also, Ki67 and Toll-like receptor 5 (TLR5) have been suggested as promising biomarkers [15, 19].

The aim of this study was to evaluate the value of p53, Ki67, and TLR5 in prediction of malignant progression of metaplasia and LGD in a matched case–control study derived from Northern and Central Finland.

## Materials and methods

### Study design and data collection

This study was a retrospective matched case–control study based on Northern and Central Finland patients. Those diagnosed with high-grade dysplasia or esophageal adenocarcinoma were included in the study from Northern Finland between January 1, 1998 and December 31, 2013, and from Central Finland January 1, 1995 and December 31, 2014. Eligible patients were identified from pathology reports. From these patients, all previous endoscopy samples were obtained along with the original diagnostic HE-slides. Clinical data was collected from the patient records. All patients with at least a single endoscopy performed more than 6 months before the diagnosis of high-grade dysplasia or adenocarcinoma, and with a biopsy sample from the esophagus were included. All endoscopies with Barrett’s esophagus or low-grade dysplasia during the 6 months before high-grade dysplasia or adenocarcinoma diagnosis were excluded from disease progression analyses.

Controls were identified from hospital archives and pathology reports, and included non-progressive Barrett’s metaplasia and non-progressive low-grade dysplasia confirmed with follow-up endoscopies performed at least 5 years after the initial diagnosis, matched by age (± 5 years) and sex to the cases.

**Fig. 1:**
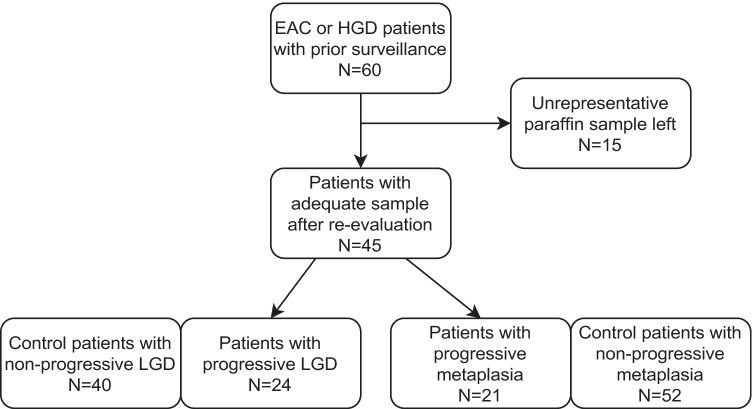
Flow chart of patient group formation

All original samples were independently re-analyzed by two gastrointestinal pathologists (JM, TJK), blinded from original diagnoses and clinical data, and compared with newly stained HE-sections to verify the quality of paraffin blocks in terms of representativeness of Barrett’s metaplasia or LGD. In case of discrepancies in diagnosis, the final diagnosis was decided with consensus among the pathologists. If the paraffin block did not contain representative tissue, the case was removed from the study. Histological diagnoses of intestinal metaplasia, indefinite for dysplasia, LGD, high-grade dysplasia (HGD), and adenocarcinoma were assigned strictly following the guidelines [20–22]. Due to overlap in the diagnoses, indefinite for dysplasia and low-grade dysplasia were combined in the final analysis. Data collection and the patient groups are presented in Fig. 1.

The study was approved by the Oulu University Hospital Ethics Committee. The need to obtain a written or oral consent from the patients was waived by the Finnish National Authority for Medicolegal Affairs (VALVIRA).

### Immunohistochemistry

Paraffin-embedded samples were sectioned and stained for p53, Ki67, and TLR5. Antigen retrieval was performed by exposure to high temperature in 800 W microwave for 2 min and 300 W for 15 min in Tris–EDTA buffer (pH 9.0). Immunostaining was performed manually with mouse antibodies against TLR5 (NBP2-24787) at a dilution of 1:75, overnight in a refrigerator, Ki-67 (Bond, Leica REF PAO230) without dilution, 60 min in room temperature, and p53 (DAKO monoclonal mouse clone DO-7), at a dilution of 1:400, 30 min in room temperature. For all antibodies, detection of the first antibody binding we used Dako REAL EnVision Peroxidase/DAB + , Rabbit/Mouse, REF K5007 (Dako, Copenhagen, Denmark). The reaction was visualized by Dako REAL™ DAB + Chromogen. As a negative control, we omitted the primary antibody and replaced the primary antibody with a non-specific mouse primary antibody isotype.

### Exposure (p53, Ki67, and TLR5 staining)

The representative tissue samples were stained with p53, Ki67, and TLR5, and the sections were scanned and digitized using Aperio AT2 (Leica Biosystems, Wetzlar, Germany). The staining was analyzed from scanned slides using Aperio ImageScope by two independent researchers (O.H. and H.H.) strictly blinded to the clinical and outcome data. The samples were placed in random order by laboratory staff to prevent any possibility of analysis-related bias.

In line with previous studies [14, 15], the following cut-off values and classifications for immunohistochemical expression patterns were used. For p53 and Ki67 only nuclear staining was evaluated:

**p53** [15, 16]**:**

1. Intensity: Loss of expression (negative), normal expression (weak to moderate intensity), overexpression (strong intensity). Intensity was considered “aberrant” if there was either loss of expression or overexpression.
2. Percentage of positive cells: 0% regarded as negative, < 15% regarded as normal, 15–40% regarded as moderate percentage, > 40% as high percentage.

**Ki67** [15]**:**

1. Percentage of positive cells: < 20% regarded as normal, 20–50% regarded as moderate percentage, > 50% as high percentage.

**TLR5** [19, 23]**:**

1. Intensity: < 2 regarded as low, ≥ 2 regarded as high (in scale from 0 to 3).
2. Percentage of positive cells: < 100% low, 100% high
3. Nuclear positivity: ≤ 80% low, > 80% high

### Statistical analyses

Baseline characteristics of progressors and non-progressors were compared using the Mann–Whitney *U* test and chi-square test as appropriate. To estimate the value of p53, Ki67, and TLR5 in neoplastic progression, generalized linear mixed model (GLMM) was used to calculate odds ratios (ORs) and 95% confidence intervals (CIs). Both cases and controls could have more than one sample collected at different time points, and a case could have more than one control; therefore, patient ID and matching number were used as random effects in GLMM. A two-sided *p* value < 0.05 was considered statistically significant. IBM SPSS Statistics 24.0 (IBM corp., Armonk, NY) was used for all statistical analyses.

## Results

Initially, cases included 60 patients, who had developed HGD or adenocarcinoma during the follow-up with a total of 367 endoscopies. Controls included 117 patients with no such progression during the follow-up with 578 endoscopies. Based on original pathology reports, there were 34 patients with progressive Barrett’s metaplasia and 26 patients with progressive low-grade dysplasia included in the cases. The age- and sex-matched controls (1–3 controls per case) included 73 patients with non-progressive Barrett’s metaplasia and 44 with non-progressive low-grade dysplasia, based on the original reports. All biopsies were screened and re-evaluated by the two expert gastrointestinal pathologists, comparing diagnostic HE-slides and new sections from the remaining paraffin samples. Adequate and representative tissue material within inclusion criteria was left in 45 cases in total of 131 samples, and 92 controls with 126 samples. Based on this re-evaluation, patients progressing to either HGD or adenocarcinoma, were divided into two groups: with diagnosis of prior LGD (progressive LGD group, 24 patients), or with diagnosis of prior metaplasia (progressive metaplasia group, 21 patients). In the progressive LGD group, re-evaluation of eventually included samples during the whole follow-up resulted with 8 adenocarcinoma, 19 HGD, 28 LGD, 14 indefinite for dysplasia, and 15 metaplasia. In the progressive metaplasia group, final samples during the follow-up included 18 adenocarcinomas, 4 HGD, and 25 metaplasia, respectively. LGD and indefinite for dysplasia groups were further combined in analyses regarding progression risk. The group selection is summarized in the flow chart (Fig. 1).

Baseline characteristics including the comparison of the case (progressive disease) and control (non-progressive disease) groups are presented in Table 1.

**Table 1:** Baseline characteristics of study groups. Presented numbers in each group are patients

	Controls	Cases		Controls	Cases	
	Non-progressive metaplasia *n* = 52	Progressive metaplasia *n* = 21	*p* value	Non-progressive low-grade dysplasia *n* = 40	Progressive low-grade dysplasia *n* = 24	*p* value
Follow-up time, years (IQR)	8.2 (6.0–11.0)	5.3 (1.2–14.6)	0.107	4.9 (1.9–8.2)	5.9 (2.1–7.7)	0.948
Endoscopies, median (IQR)	3 (1–4)	4 (1–5)	0.362	5 (3–6)	6 (3–13)	0.211
Stained samples	54	47		72	84	
^1^Age, years, median (IQR)	66.8 (61.1–71.5)	64.3 (56.5–69.8)	0.603	71.2 (65.0–77.4)	68.5 (60.2–78.4)	0.333
Sex, men	40 (77)	12 (57)	0.152	36 (90)	22 (92)	1.000
^1^BMI	28.0 (25.5–29.7)	27.5 (25.4–31.7)	0.572	26.5 (24.0–28.1)	26.4 (24.1–30.9)	0.388
^2^Esophagitis, n (%)	22 (41)	17 (68)	0.024	24 (52)	18 (46)	0.580
^2^Reflux symptoms	37 (69)	23 (92)	0.023	33 (72)	28 (72)	0.995
^2^Regular NSAID usage	11 (20)	7 (28)	0.452	8 (17)	11 (28)	0.233
^2^Prior PPI usage	40 (74)	21 (84)	0.328	37 (80)	28 (72)	0.349

^1^At last endoscopy

^2^Out of evaluated metaplasia and LGD samples (not including end-point samples of HGD or adenocarcinoma). Samples with missing information were excluded

## p53, Ki67, and TLR5 as biomarkers for progression in low-grade dysplasia

### p53 intensity and percentage of positive cells

**Fig. 2:**
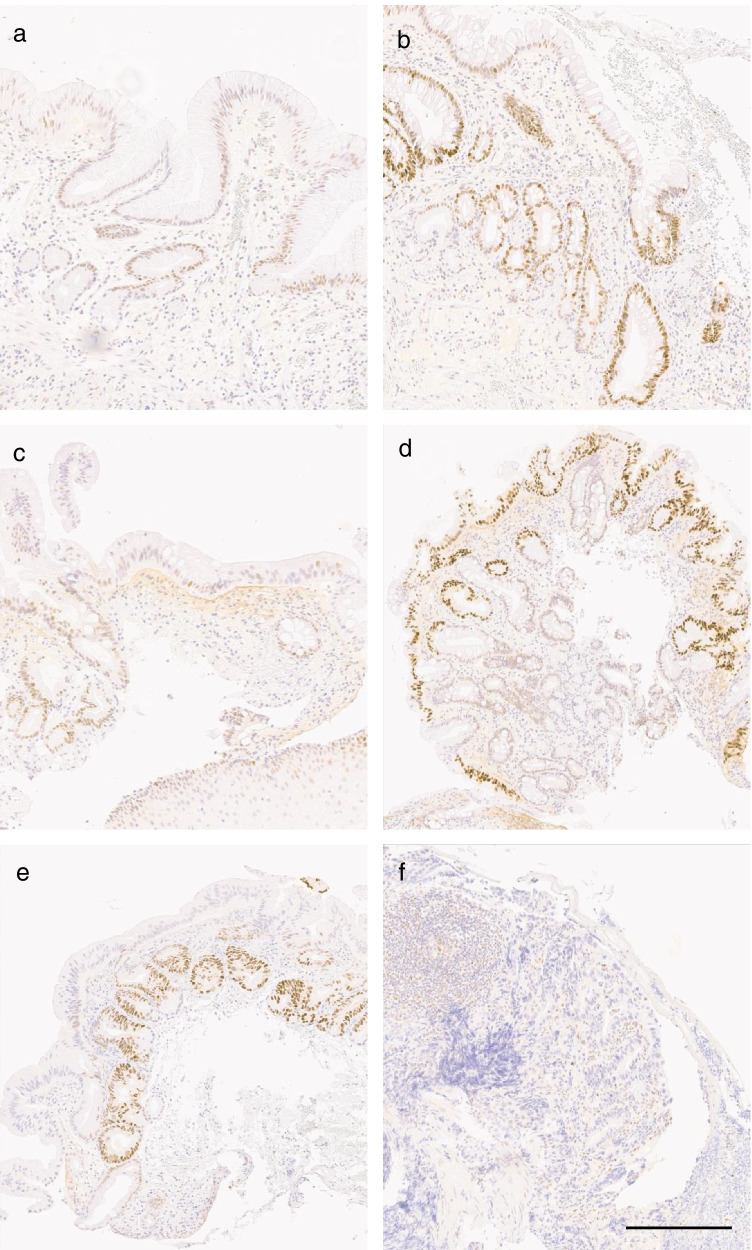
p53 expression patterns: **a** normal expression in non-progressive Barrett’s metaplasial; **b** over expression in progressive Barrett’s metaplasia; **c** normal expression in non-progressive low-grade dysplasia; **d** and **e** over expression in progressive low-grade dysplasia; and **f** loss of expression in high-grade dysplasia. Scale bar 300um

p53 overexpression (high intensity of expression) was common in cases with progressive LGD. Of 39 LGD samples, such overexpression was detected in 22/39 (56%), and in 15/24 (63%) patients. Numbers in non-progressive LGD controls were 8/46 (17%; samples) and 7/40 (18%; patients), respectively. p53 overexpression resulted in OR 6.7 (95% CI 1.8–24.6) for progression, Fig. 2, Table 2. Percentage of p53 positive cells showed no differences between progressive cases and non-progressive LGD controls. High percentage was more common in LGD samples when compared to metaplasia samples (Table 2; *p* < 0.001; Fisher exact test.).

**Table 2:** Progression risk of non-dysplastic Barrett’s metaplasia and low-grade dysplasia patients related to expression of p53, Ki67, and TLR5. Figures indicate the numbers of samples. Percentages are indicated in parentheses

	Non-progressive Barrett’s metaplasia	Progressive Barrett’s metaplasia	OR (95% CI)	Non-progressive low-grade dysplasia	Progressive low-grade dysplasia	OR (95% CI)
Samples	*n* = 54	*n* = 25		*n* = 46	*n* = 39	
p53 intensity						
Normal expression*	54 (100)	24 (96)	1 (Ref)	38 (83)	17 (44)	1 (Ref)
Aberrant expression**	0 (0)	1 (4)	Non est	8 (17)	22 (56)	6.7 (95% CI 1.8–24.6)
Overexpression**	*0 (0)*	*0 (0)*		*8 (17)*	*22 (56)*	
*Loss of expression*	*0 (0)*	*1 (4)*		*0 (0)*	*0 (0)*	
p53 percentage						
< 15%	40 (74)	13 (52)	1 (Ref)	4 (9)	1 (3)	1 (Ref)
15–40%	12 (22)	11 (44)	2.69 (0.83–8.69)	19 (41)	11 (28)	1.96 (0.11–35.10)
> 40%	2 (4)	1 (4)	1.73 (0.10–29.16)	23 (50)	27 (69)	4.54 (0.27–75.3)
Ki67						
< 20%	25 (46)	14 (56)	1 (Ref)	1 (2)	4 (10)	1 (Ref)
20–50%	24 (44)	9 (36)	0.65 (0.20–2.06)	14 (30)	12 (31)	0.21 (0.01–3.80)
> 50%	5 (9)	2 (8)	0.69 (0.09–5.22)	31 (67)	23 (59)	0.17 (0.01–2.86)
TLR5						
Intensity < 2	41 (76)	20 (80)	1 (Ref)	14 (38)	17 (44)	1 (Ref)
Intensity ≥ 2	13 (24)	5 (20)	0.83 (0.22–3.09)	32 (62)	22 (56)	0.55 (0.17–1.80)
Percentage < 100%	38 (70)	12 (48)	1 (Ref)	18 (39)	10 (26)	1 (Ref)
Percentage 100%	16 (30)	13 (52)	2.73 (0.88–8.49)	28 (61)	29 (74)	2.06 (0.60–7.07)
Nuclear positivity ≤ 80%	43 (80)	19 (76)	1 (Ref)	18 (39)	23 (59)	1 (Ref)
Nuclear positivity > 80%	11 (20)	6 (24)	1.23 (0.34–4.44)	28 (61)	16 (41)	0.47 (0.15–1.47)

Odds ratios were calculated using generalized linear mixed model where cases and controls were matched by age and sex. Studied immunohistochemical markers in premalignant (metaplasia and low-grade dysplasia) samples were based on HE-diagnoses of two expert gastrointestinal pathologists stratified by disease progression status

^*^Normal expression was based on weak to moderate intensity score

^*^*Aberrant expression in p53 intensity included both loss of expression and overexpression

^***^Overexpression was based on the presence of high intensity score

### Ki67 percentage of positive cells

No significant differences were observed in Ki67 expression between progressive cases and non-progressive LGD controls. Moderate and high percentage were common in LGD when compared to metaplasia samples (Table 2).

### TLR5 intensity, percentage of positive cells and nuclear positivity

No significant differences were observed in either TLR5 intensity, percentage of positive cells or nuclear positivity between progressive cases and non-progressive LGD controls (Table 2).

## p53, Ki67, and TLR5 as biomarkers for progression in Barrett’s metaplasia

### p53 intensity and percentage of positive cells

p53 overexpression was never observed in either progressive cases or non-progressive Barrett’s metaplasia controls. A single metaplasia sample in progressive cases group showed loss of expression (Table 2). Percentage of positive cells showed no differences between progressive cases and non-progressive Barrett’s metaplasia controls. High percentage was rare (Table 2).

### Ki67 percentage of positive cells

No significant differences were observed in Ki67 expression between progressive cases and non-progressive Barrett’s metaplasia controls. High percentage was rare in both groups (Table 2).

### TLR5 intensity, percentage of positive cells, and nuclear positivity

No significant differences were observed in either TLR5 intensity, percentage of positive cells, or nuclear positivity between progressive cases and non-progressive Barrett’s metaplasia controls. Overall, lower TLR5 expression was observed in metaplasia samples when compared to low-grade dysplasia samples (Table 2).

## Prevalence of aberrant p53 expression in metaplasia-dysplasia-adenocarcinoma sequence

**Fig. 3:**
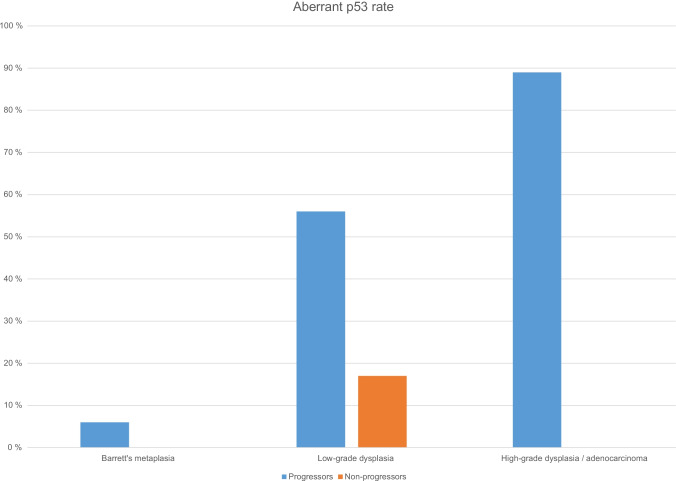
Aberrant p53 rate in metaplasia-dysplasia-adenocarcinoma sequence

When including all studied samples of HGD and adenocarcinoma, and detected Barrett’s metaplasia samples in LGD groups, aberrant (either loss of expression or overexpression) p53 expression increased during follow-up in patients with progressive disease when compared to non-progressive patients (Fig. 3). In the progressive LGD group, p53 overexpression was detected in 18/24 (75%) and loss of expression in 4/24 (17%) of HGD and adenocarcinoma samples (end-point), and the numbers in progressive Barrett’s metaplasia group were 9/21 (43%) and 9/21 (43%), respectively. When analyzing p53 expression in Barrett’s metaplasia samples in the progressive LGD group, aberrant expression was observed in 6 metaplasia samples (5 overexpression, 1 loss of expression) in progressors compared to 0 aberrant samples in non-progressors. Overall, p53 was analyzed from 114 metaplasia samples (35 from progressor cases and 79 from non-progressor controls), with 7 aberrant expression, all in progressor cases.

## Discussion

In this case–control study of progressive and non-progressive Barrett’s metaplasia and dysplasia patients, p53 associated with a high risk of progression. The previously suggested markers Ki67 and TLR5 were not associated with disease progression.

The main strength of this study is the relatively large sample size of esophageal adenocarcinomas and high-grade dysplasia from two geographical areas, out of which the final patient cohort was identified. Endoscopies were performed in community setting under routine care, where strict sampling protocol was probably not always used. Therefore, these results can be considered as “real-life.” All samples were re-cut and stained, evaluated blindly by two expert gastrointestinal pathologists and compared with the original diagnostic slides to confirm or revise the original diagnosis. The new sections also confirmed that the paraffin block contained adequate tissue sample for immunohistochemical analyses. With this strict screening, the final cohort of progressive and non-progressive metaplasia and dysplasia patients can be considered reliable. All samples with insufficient tissue, non-confirmed diagnoses and shorter than a 6-month interval to high-grade dysplasia or adenocarcinoma diagnosis were excluded. p53 and Ki67 are routine stainings in surgical pathology laboratories and therefore generalization of these results can be made. However, there are also some limitations. With the retrospective design, only patients with prior biopsies from Barrett’s esophagus could be included, representing a minority of all diagnosed esophageal adenocarcinomas in the region. However, the study is still generalizable to the patients presenting with Barrett’s esophagus without dysplasia or with low-grade dysplasia, i.e., the population where measures to mitigate the risk of malignant transformation would be necessary. Endoscopies were performed slightly more often in progressive cases. There is a possibility that in progressive cases there were some clinical suspicion or difference in clinical presentation that we are not aware of. However, since number of endoscopies showed no statistical difference, this confounder is probably limited.

Commonly known tumor suppressor gene p53 has been identified as an important marker for progression in Barrett’s epithelium. In a recent systematic review and meta-analysis, a total of 15 studies assessing the potential of p53 was reported [24]. Of these, 7 included patients with low-grade dysplasia diagnosis and others either did not report exact histology or consisted of non-dysplastic Barrett’s metaplasia [24]. All these studies had relatively low number of patients progressing to high-grade dysplasia or carcinoma: in case–control studies a total of 209 progressors and in cohort studies 28 progressors were identified [24]. In case–control studies, OR of 3.84 (95%CI 2.79–5.27) and in cohort studies RR of 17.3 (95%CI 9.35–32.1) was reported for abnormal p53 expression. A recent large retrospective cohort study including both non-dysplastic and dysplastic Barrett’s metaplasia showed high prognostic potential of p53 staining also in real-world setting [17]. In the current study, we observed somewhat higher OR (6.7, 95% 1.8–24.6), although confidence interval overlapped with those reported previously. Of these previous studies, Kastelein et al. is the largest with 49 progressors (34 with prior low-grade dysplasia and 15 with metaplasia), and reported RR as high as 11.2-fold in case of dysplasia and aberrant p53 expression [16]. They, however, do not state whether biopsies prior to end-point were excluded, and whether multiple samples per patient were used. In our study, last 6 months prior to end-point were excluded. In addition to endoscopy and immunohistochemical analysis, novel methods such as Cytosponge have been developed [18]. Cytosponge-based p53 overexpression has been suggested to be a strong risk factor for malignant progression, although positive predictive value is relatively low limiting its clinical use [18]. Although with limited evidence, ESGE guidelines suggest surveillance even in non-dysplastic Barrett’s esophagus except in cases with very short metaplasia area [12]. Therefore, based on current and previous evidence, it is well justified to conduct surveillance in patients with aberrant p53 expression also without dysplasia. It could be even possible to offer endoscopic treatment due to high progression risk, although this change in practice needs further evidence. Expert confirmed LGD should undergo endoscopic treatment, and based on accumulating evidence especially those with aberrant p53 expression could benefit from aggressive approach aiming for complete eradication [12].

Biomarkers other than p53 suggested for evaluation of Barrett’s lesions include Aspergillus oryzae lectin, cyclin A and D, alpha-methylacyl-CoA racemase, Ki67, and TLR5 [15, 19, 25]. None of these have gained wide acceptance and have not been suggested for routine use. Ki67, which is present in proliferating cells and absent in resting cells [26], was associated with malignant progression in a case–control study of dysplastic Barrett with 27 progressors [15]. We did not observe similar association as Ki67 did not show any predictive value. Also TLR5, a receptor recognizing bacterial flagellin and activating immunity reaction, is overexpressed in metaplasia-dysplasia-carcinoma sequence [19], showed higher expression in LGD when compared non-dysplastic Barrett’s esophagus, but seems not to be a potential marker for progression. However, sample size and limited power can result with some missed positive associations for both Ki67 and TLR5, and replication studies are needed.

This study is one of the largest assessing progressive Barrett’s esophagus, both with and without dysplasia, and has important clinical implications. Currently, there are no clear evidence-based guidelines regarding screening or surveillance of Barrett’s esophagus without dysplasia [12]. In case of low-grade dysplasia, current European Society of Gastrointestinal Endoscopy guidelines suggest surveillance interval of 6 months, possibly prolonged to 1 year, but if LGD is found in subsequent samples, removal of the diseased mucosa should be offered [12]. Still, when based only on histological diagnosis, a 10-year progression rate of LGD is around 15% even after consensus of multiple pathologists [15]. On the other hand, endoscopic treatment is associated with significant adverse events, including a 5% stricture rate [27]. With more accurate risk stratification individuals with high risk can be treated, accepting the possibility of adverse events, while low-risk patients could continue endoscopic follow-up. Based on this study, readily available p53 staining should be included as a routine staining in Barrett’s esophagus and LGD. If aberrant expression of p53 is detected, endoscopic treatment of dysplastic and metaplastic mucosa is indicated.

## Conclusion

In conclusion, aberrant expression of p53 in LGD is associated with high risk of progression to HGD or carcinoma. Routine staining of p53 is indicated in expert confirmed LGD, and aberrant expression of p53 in dysplastic Barrett’s epithelium is an indication for endoscopic treatment of the diseased mucosa.

## Declarations

The study was conducted according to the guidelines of the Declaration of Helsinki and was approved by the hospital districts. Use of the patient data were approved by the Ethics Committee (EETTMK: 81/2008). The need for written informed consents was waived by National Authority for Medicolegal Affairs (VALVIRA) due to its retrospective nature.
